# Risk of Osteoporosis-Related Fracture in Children and Adolescents with Intellectual Disability

**DOI:** 10.3390/medicina61101761

**Published:** 2025-09-29

**Authors:** Jeong Rae Yoo, Jeong Ho Kang, So Young Lee, Jun Hwan Choi, Hyun Jung Lee

**Affiliations:** 1 Department of Internal Medicine, Jeju National University Hospital, College of Medicine, Jeju National University, Aran 13-gil 15, Jeju 63241, Republic of Korea; mdyoojr@jejunu.ac.kr; 2 Department of Emergency Medicine, Jeju National University Hospital, College of Medicine, Jeju National University, Aran 13-gil 15, Jeju 63241, Republic of Korea; siva123456@jejunu.ac.kr; 3 Department of Physical Medicine and Rehabilitation, Jeju National University Hospital, College of Medicine, Jeju National University, Aran 13-gil 15, Jeju 63241, Republic of Korea; bluelsy900@hanmail.net (S.Y.L.); miraerojh0728@gmail.com (J.H.C.)

**Keywords:** intellectual disability, osteoporosis, fractures, bone, adolescent, child, cohort studies

## Abstract

*Background and Objectives*: Children and adolescents with intellectual disability (ID) experience substantial health disparities, yet their skeletal health has been overlooked. Early-onset osteoporosis and fracture remain underrecognized in this population. Hence, this study assessed the risk of osteoporosis with concomitant fracture in this population using nationwide cohort data. *Materials and Methods*: This population-based retrospective cohort study examined data from South Korea’s National Health Insurance Service-National Health Information Database (2004–2021). In all, 75,790 individuals with ID and 922,921 control individuals aged 2–18 years were included. The primary outcome was osteoporosis with concomitant fracture occurring within 1 year before or after the osteoporosis diagnosis. The secondary outcome was osteoporosis with a pathological fracture. *Results*: The ID group had a significantly higher risk of developing osteoporosis with concomitant fracture than the control group (unadjusted hazard ratio [HR], 6.821; 95% confidence interval [CI], 5.065–9.187; *p* < 0.001). This association remained significant after adjusting for demographic factors and medical comorbidities as a composite variable (HR, 4.385; 95% CI, 3.080–6.245; *p* < 0.001) and after additional adjustment for cerebral palsy (HR, 3.331; 95% CI, 2.252–4.926; *p* < 0.001). Subgroup analyses showed stronger associations in males (HR, 7.597), younger ages (7–11 years: HR, 9.501), and rural areas (HR, 8.882). *Conclusions*: Children and adolescents with ID have a high risk of osteoporosis with concomitant fracture. Early assessment and targeted strategies are needed to promote bone health in this population.

## 1. Introduction

Intellectual disability (ID) is a neurodevelopmental disorder marked by deficits in cognitive functioning and adaptive behavior, affecting approximately 1–3% of children and adolescents globally [1]. Individuals with ID experience considerable health disparities relative to the general population, including elevated rates of chronic conditions, mental health disorders, and emerging concerns about skeletal health [2,3].

Osteoporosis, a progressive skeletal disease that compromises bone density and structural integrity, has traditionally been a focus of geriatric research [4]. Bone mass accrual occurs rapidly during childhood and adolescence, with more than 50% of skeletal formation occurring in the teenage years [5]. Bone mass increases rapidly within 6 months following the adolescent growth spurt, continuing until peak levels are typically reached in late adolescence or early adulthood [5,6]. Early bone mass acquisition significantly influences lifelong skeletal health; epidemiological studies suggest that a 10% increase in peak bone mass at the population level decreases the risk of fracture later in life by 50% [5].

Children and adolescents with ID have multiple risk factors for compromised bone health. Several comorbid conditions, including epilepsy, cerebral palsy (CP), and various genetic conditions, directly affect bone metabolism [7,8,9]. The medications required to manage these conditions, particularly antiepileptic drugs (AEDs), can impair calcium absorption, increase vitamin D metabolism, and directly affect bone cell function, leading to reduced bone mineral density [10]. Additionally, reduced physical activity due to motor impairments—commonly seen in children with ID, particularly those with comorbid CP—limits the mechanical loading necessary for bone formation [11]. Many individuals with ID also face nutritional challenges due to feeding difficulties, selective eating behaviors, or medication-related appetite changes, which further compromise their bone health [12]. Although prior studies have linked ID with reduced bone mineral density and higher fracture risk [3,13], and while recent modeling approaches have enhanced fracture risk prediction in general populations [14], comprehensive research focusing on children and adolescents with ID remains limited, underscoring the urgent need for population-specific investigation.

The National Health Insurance Service—National Health Information Database (NHIS-NHID) of South Korea provides a unique opportunity to examine this issue. The NHIS-NHID covers approximately 98% of the South Korean population and offers extensive longitudinal data on healthcare utilization, diagnosis, and treatment. To our knowledge, no large-scale study has leveraged this resource to investigate osteoporosis risk in Korean children with ID, leaving a significant gap in the understanding and management of bone health in this population.

Given that the diagnosis of osteoporosis in children typically requires the presence of clinically significant fractures in addition to bone mineral density criteria and that dual-energy X-ray absorptiometry (DXA) is rarely performed in routine pediatric care [15,16], we used a composite outcome of ‘osteoporosis with a concomitant fracture’ to more accurately reflect clinically meaningful bone fragility in this population.

Using the NHIS-NHID data, this study aimed to (1) assess the risk of osteoporosis with concomitant fracture between children and adolescents with and without ID and (2) identify associated risk factors in this population. We hypothesized that children and adolescents with ID have a significantly higher risk of osteoporosis with concomitant fracture than those without ID and that this risk varies according to specific clinical and demographic factors.

## 2. Materials and Methods

### 2.1. Study Design and Data Source

This retrospective cohort study utilized data from the NHIS-NHID, spanning 1 January 2004 to 31 December 2021. The NHIS-NHID provides nationwide medical claims data for most South Koreans, containing detailed records on diagnoses, treatments, and demographic information. All diagnoses in the database are coded using the International Classification of Diseases, 10th Revision (ICD-10), which South Korea adopted in 2002. The study protocol was approved by the Jeju University Hospital (JEJUNUH 2024-01-017), with a waiver of informed consent given the retrospective nature of the study and use of anonymized data. All procedures performed in studies involving human participants were in accordance with the ethical standards of the institutional and/or national research committee and with the 1964 Helsinki declaration and its later amendments or comparable ethical standards.

### 2.2. Population Selection

From the initial dataset, 856 individuals with incomplete demographic profiles, e.g., individuals without standard medical insurance coverage, including non-citizens and those without permanent housing, were excluded.

The ID group included individuals with ID who were enrolled continuously from 2004 to 2021. New participants entered the cohort annually at their first ID diagnosis. The inclusion criteria were (1) at least two diagnoses of F70-F79.9 and (2) age of 2–18 years. From an initial group of 75,976 individuals, we excluded 186 with osteoporosis, resulting in 75,790 participants.

The control group was selected from the 2004 database only, with 1:10 matching by sex and age (n = 923,058). The inclusion criteria were (1) no diagnosis of ID (F70–F79.9) and (2) age of 2–18 years. After excluding 137 individuals with osteoporosis before 2004, the final control group comprised 922,921 participants who remained free of ID diagnoses throughout the entire study period.

**Figure 1:**
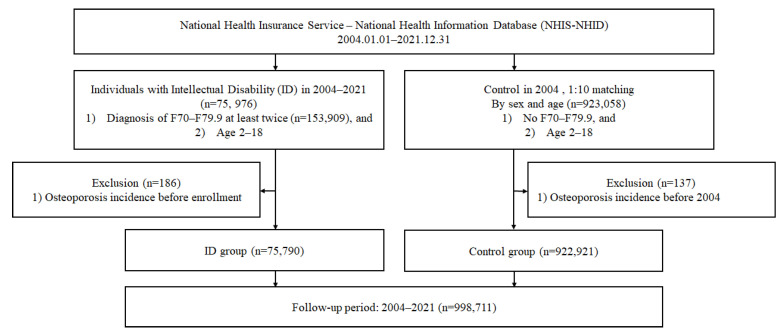
Flowchart depicting the selection process of the study population.

Follow-up continued from 1 January 2004 until the earliest of the following: death, loss to follow-up, or the study endpoint (31 December 2021). However, owing to the use of a complete claims database, no individual was lost to follow-up during the study period. Owing to the fixed enrolment of controls in 2004 and dynamic inclusion of ID cases upon diagnosis, the mean follow-up duration was longer in the control group (Figure 1).

### 2.3. Outcome Measures

The primary outcome was osteoporosis with a concomitant fracture, defined as at least one medical claim with ICD codes M80–M82 (Table S1 in the Supplement S1); this study included patients with a fracture diagnosis occurring within 1 year before or after the osteoporosis diagnosis—this time window was selected to capture fractures temporally related to bone health impairment. The 1-year window was chosen to capture temporally related fractures while reflecting clinical practice patterns where osteoporosis evaluation in pediatric populations typically follows fracture occurrence. The secondary outcome was osteoporosis with pathological fractures (M80), representing cases in which fractures were directly attributed to bone fragility.

### 2.4. Explanatory Variables

Demographic variables included age group (categorized as 2–6, 7–11, and 12–18 years), sex, and age at first ID diagnosis. ID severity was classified as mild, moderate, severe, profound, or others/unspecified based on corresponding ICD-10 codes. Socioeconomic status was categorized into five groups: medical aid recipients representing no income, low income (≤7th), lower-middle (8th–13th), upper-middle (14th–16th), and high income (≥17th). The residential area was classified as urban or rural based on municipal definitions.

Medical comorbidities included hypertension, diabetes, renal failure, epilepsy treated with antiepileptic drug (AED), rheumatoid arthritis, hypothyroidism, hyperthyroidism, hyperparathyroidism, chromosomal abnormalities, and CP; these were identified using relevant ICD-10 codes from the NHIS-NHID database. Healthcare utilization was assessed through participation in national health checkup programs.

### 2.5. Statistical Analysis

We compared baseline characteristics between groups using chi-squared tests for categorical variables and *t*-tests for continuous variables. Missing data were minimal: socioeconomic status (1.27%, n = 12,666) and residential area (0.013%, n = 131). These cases were included in descriptive and univariate analyses but excluded from multivariate analyses when relevant variables were included as covariates.

The incidence of osteoporosis with concomitant fracture was calculated per 1000 person-years. Cumulative incidence curves were generated using the Kaplan–Meier method and compared using log-rank tests. We conducted Cox regression analyses for our primary outcome, including univariate analysis for all variables and multivariate analysis with two models: Model 1 adjusted for demographics and medical comorbidities (composite), and Model 2 further adjusted for CP. For subgroup analyses involving CP, we acknowledged that comparisons might be limited by low event rates in control subgroups, potentially resulting in non-estimable hazard ratios due to zero events in specific strata.

The secondary outcome (pathological fractures, M80) was analyzed with two models: Model 1, adjusted for demographics and medical comorbidities (composite), and Model 2, with additional adjustment for CP. Subgroup analyses were conducted by sex, age group, socioeconomic status, medical comorbidities, and CP status. All analyses used SAS 9.4 (SAS Institute, Cary, NC, USA), with significance set at *p* < 0.05. This study followed the STROBE reporting guideline for cohort studies.

## 3. Results

### 3.1. Baseline Characteristics

The ID group was followed for 10.9 ± 4.9 years, while the control group had a follow-up period of 18.0 ± 0.1 years. Males were predominant in both groups (64.7% vs. 64.0%). The mean age at first ID diagnosis was 10.3 ± 4.6 years (Table 1). The ID group had higher proportions of medical aid recipients (18.9% vs. 3.1%) and rural residents (34.4% vs. 31.1%). By severity, mild ID was most common (41.1%), followed by moderate (29.9%), severe (15.0%), and profound ID (4.5%). The prevalence of medical comorbidities was significantly higher in the ID group than in the control group (23.8% vs. 1.6%). The detailed clinical characteristics of the participants, including the prevalence of individual medical conditions, are presented in Table S2 in Supplement S1. The most common comorbidities in the ID group were AED-treated epilepsy (17.46% vs. 0.25%) and CP (8.67% vs. 0.05%). The ID group had lower health screening rates than the control group (22.3% vs. 52.2%).

### 3.2. Incidence and Risk of Osteoporosis with Concomitant Fracture

**Figure 2:**
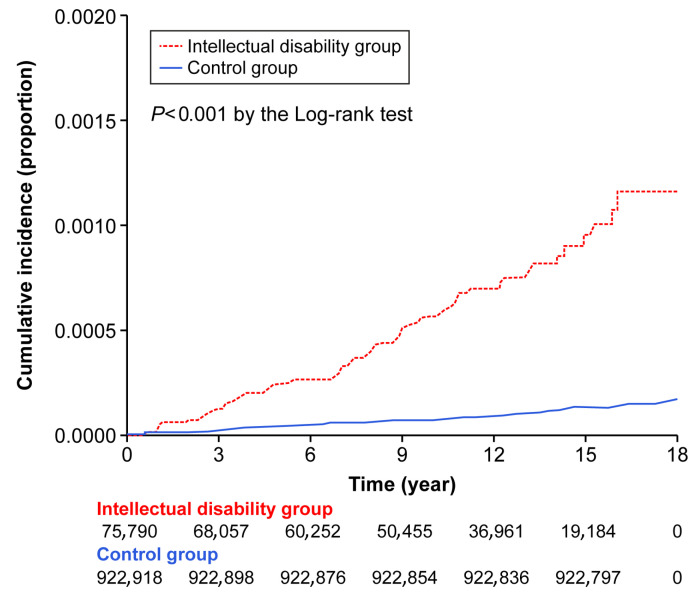
Cumulative incidence of osteoporosis with concomitant fracture in the intellectual disability and control group.

We identified 60 cases of osteoporosis with concomitant fracture in the ID group and 218 in the control group, with incidence rates of 0.072 vs. 0.013 per 1000 person-years, respectively (Table S3 in Supplement S1). The cumulative incidence was significantly higher in the ID group (log-rank test, *p* < 0.001) (Figure 2).

The ID group exhibited a significantly elevated risk of osteoporosis with concomitant fracture (unadjusted hazard ratio [HR], 6.821; 95% confidence interval [CI], 5.065–9.187). This association remained robust after adjustment for demographics and medical comorbidities as a composite variable (Model 1: HR, 4.385; 95% CI, 3.080–6.245), and further adjustment for cerebral palsy (Model 2: HR, 3.331; 95% CI, 2.252–4.926) (Table 2).

Several demographic and clinical factors were associated with increased risk. In the fully adjusted model (Model 2), older age (12–18 years: HR, 2.179; 95% CI, 1.603–2.963) and female sex (HR, 1.265; 95% CI, 0.996–1.606) showed elevated risk, although the latter did not reach conventional significance. A clear socioeconomic gradient was observed, with progressively lower risk in higher income groups than in medical aid recipients. CP was independently associated with a markedly increased risk (HR, 6.523; 95% CI, 3.777–11.265).

The association between ID and osteoporosis with pathological fractures was even stronger. The cumulative incidence was significantly higher in the ID group (*p* < 0.001 by log-rank test) (Figure S1 in Supplement S1). The unadjusted HR was 7.420 (95% CI, 5.338–10.313), and the association remained significant after adjusting for demographics and comorbidities (HR, 4.730; 95% CI, 3.186–7.021), further adjusting for CP (HR, 3.439; 95% CI, 2.207–5.358) (Table S4 in Supplement S1).

Fracture site distribution differed significantly between groups, with the ID group showing higher rates of femur and upper extremity fractures, while the control group had higher rates of forearm and wrist fractures (Table S5 in Supplement S1).

### 3.3. Subgroup Analyses

The association between ID and risk of osteoporosis with concomitant fracture varied across the subgroups (Table 3). The sex-stratified analysis revealed a stronger association in males (HR, 7.597; 95% CI, 5.225–11.045) than in females. When stratified by age, the strongest associations were observed in children aged 7–11 years (HR, 9.501; 95% CI, 5.479–16.476) and 2–6 years (HR, 8.188; 95% CI, 4.354–15.399), with a relatively lower magnitude in the 12–18-year group (HR, 4.859; 95% CI, 3.157–7.479). Rural residents exhibited a higher relative risk (HR, 8.882; 95% CI, 5.218–15.117) than urban residents. The presence of medical comorbidities did not substantially modify the association. Notably, in individuals without CP, ID was significantly associated with increased risk (HR, 4.773; 95% CI, 3.339–6.822). In the CP subgroup analysis, no fracture events occurred in the control group with CP (n = 417), making direct risk comparison non-estimable.

## 4. Discussion

This nationwide population-based cohort study demonstrates that children and adolescents with ID have a markedly elevated risk of osteoporosis with concomitant fracture. In the primary analysis, the risk was more than six times higher than that in the control group (HR, 6.821; 95% CI, 5.065–9.187; incidence rate: 0.072 vs. 0.013 per 1000 person-years). This association remained robust after adjusting for demographic factors, medical comorbidities, and CP. These findings underscore the importance of implementing timely screening and targeted prevention strategies to address bone health disparities. Given the particularly high risk in children with CP and those receiving antiepileptic therapy, these subgroups may warrant enhanced clinical surveillance for bone health deterioration.

Multivariate analysis identified several important risk factors. Age was a significant determinant, with older age groups exhibiting progressively higher risk than younger ones. Female sex was associated with increased risk, although this association did not reach conventional significance. A strong socioeconomic gradient was observed, with all income groups showing significantly lower risk compared to medical aid recipients.

Medical comorbidities significantly increased the risk, emphasizing the need for comprehensive medical management. These conditions may impair bone health through distinct mechanisms—for example, diabetes alters bone microarchitecture [17], renal dysfunction disrupts calcium homeostasis [18], antiepileptic drugs induce vitamin D deficiency [10], thyroid hormones affect bone turnover [19], and chronic inflammation in rheumatoid arthritis contributes to systemic bone loss [20].

Although osteoporotic fractures are rare in the general pediatric population, the disproportionately high incidence in children with ID—particularly at such young ages—is concerning. Preventing fractures during childhood, especially in those with underlying skeletal vulnerability, may reduce the risk of future fractures, preserve mobility, and improve long-term functional independence and quality of life [21].

Subgroup analyses revealed distinct patterns. While older age was a general risk factor, the relative risk associated with ID was the most pronounced in middle childhood (7–11 years, HR: 9.501) and early childhood (2–6 years, HR: 8.188), with a lower magnitude in adolescence (12–18 years, HR: 4.859). This early peak may be attributable to developmental delays, early-onset comorbidities (e.g., epilepsy, chromosomal abnormalities), and insufficient physical activity during critical periods of bone mass accrual [10,22]. The relative impact of ID was greater in males (HR, 7.597 vs. 5.766), potentially reflecting sex-specific hormonal vulnerabilities such as hypogonadism or hypoandrogenism in males with ID [22,23].

In children with ID but without CP, the risk of osteoporosis with concomitant fracture remained significantly elevated (HR, 4.773; 95% CI, 3.339–6.822), indicating an independent effect of ID. Because no fractures occurred in the control group with CP, direct comparisons could not be made. However, the known effects of CP on bone metabolism and mobility suggest that its co-occurrence with ID may confer additional risk [24], although this could not be formally quantified in this study.

From a public health perspective, these findings underscore the urgent need for early fracture prevention strategies tailored to this vulnerable population. Given that osteoporosis in children is typically diagnosed only in the presence of clinically significant fractures, and DXA is rarely performed in routine pediatric care [15,16], our composite outcome of osteoporosis with concomitant fracture reflects a pragmatic and clinically relevant indicator of bone fragility.

These findings also highlight the cumulative impact of medical comorbidities, socioeconomic disadvantage, and sex-specific biological factors [22,25,26]. Potential interventions include monitoring of thyroid function, optimization of medication regimens that affect bone metabolism, and comprehensive health management for children with ID [21,27]. The strong socioeconomic gradient suggests a need for targeted support to low-income groups, including enhanced access to preventive care and nutrition [12,26,28]. Early screening during childhood is essential, as bone mass acquired during this period largely determines lifelong skeletal strength [5,6].

Currently, there are no established pediatric guidelines recommending routine bone health screening for children with ID. For example, Korean osteoporosis guidelines focus on adults, especially postmenopausal women and older men, with no recommendations for this population [29]. Our findings highlight the need to expand existing guidelines to include screening and prevention strategies for bone health in children with ID. Additionally, in our secondary analysis, ID was also strongly associated with pathological fractures (adjusted HR, 3.439; 95% CI, 2.207–5.358), further reinforcing the link between ID and compromised bone integrity [25].

Our findings align with those of prior studies from Western countries, including large-scale UK and German cohorts, which also reported elevated fracture risk in individuals with ID across the lifespan [25,30]. Notably, we observed higher hazard ratios and more pronounced subgroup differences among males and younger children, which were less consistently reported in previous studies. These differences likely reflect our specific focus on osteoporosis-related fractures rather than all fractures, capturing more clinically significant skeletal fragility events. Additionally, variations in healthcare systems, environmental exposures, and sociocultural factors between populations may contribute to these discrepancies.

Future studies should incorporate bone mineral density measurements, pubertal staging, and biochemical bone markers to clarify mechanisms and validate fracture risk models. Intervention trials are needed to identify effective prevention strategies and optimize bone health in children with ID, particularly those with multiple risk factors.

### Limitations and Strengths

This study has several limitations. First, osteoporosis and fractures were identified using diagnostic codes from claims data, not clinical or radiographic evaluation, which may have led to misclassification [31]. However, requiring the co-occurrence of fracture and osteoporosis codes likely improved specificity. Second, the study design involved different enrollment strategies for the two groups: controls were enrolled at a fixed time point (2004) while ID cases entered the cohort dynamically upon diagnosis. Although Cox proportional hazards models account for different follow-up durations through person-time analysis, this design feature should be acknowledged. Third, we lacked data on important lifestyle and clinical factors such as physical activity, nutritional status, vitamin D levels, and corticosteroid use. Fourth, our study could not separately identify specific causes of ID, including Down syndrome, which is known to have distinct bone health characteristics. Future studies should examine bone health outcomes in syndrome-specific populations. Nevertheless, our comprehensive adjustment for medical comorbidities and socioeconomic factors captured some of these unmeasured confounders indirectly. Despite these limitations, this study’s strengths include its nationwide scope, large sample size, 18-year follow-up period, and use of a clinically relevant composite outcome that reflects real-world diagnostic practices. To our knowledge, this study represents the largest population-based investigation of osteoporosis-related fracture risk in children and adolescents with ID, providing crucial evidence to guide clinical practice and health policy.

## 5. Conclusions

This nationwide cohort study demonstrates that children and adolescents with ID are at an increased risk of developing osteoporosis-related fractures, especially in early childhood. Healthcare providers should implement bone health screening and preventive strategies tailored to this population. Interventions targeting low-income groups are also needed to improve access to nutrition and essential care. Future studies incorporating bone mineral density measurements, biochemical markers, and interventional approaches are warranted to establish evidence-based pediatric bone health guidelines for this high-risk population.

## Figures and Tables

**Table 1:** Baseline demographic and socioeconomic characteristics of participants.

Characteristics	Total	Control Group	Intellectual Disability Group	*p*-Value
	998,711 (100%)	922,921 (92.4%)	75,790 (7.6%)	
Sex				<0.001
Female	359,507 (35.997)	332,751 (36.054)	26,756 (35.303)	
Male	639,204 (64.003)	590,170 (63.946)	49,034 (64.697)	
Severity of ID				<0.001
Borderline/mild	31,167 (3.121)	NA	31,167 (41.123)	
Moderate	22,691 (2.272)	NA	22,691 (29.939)	
Severe	11,348 (1.136)	NA	11,348 (14.973)	
Profound	3431 (0.344)	NA	3431 (4.527)	
Others/unspecified	7153 (0.716)	NA	7153 (9.438)	
Age at first ID diagnosis				<0.001
(mean ± SD)	9.440 ± 4.740	9.371 ± 4.748	10.28 ± 4.568	
Age group				<0.001
2–6 years	323,217 (32.363)	303,480 (32.883)	19,737 (26.042)	
7–11 years	323,534 (32.395)	299,533 (32.455)	24,001 (31.668)	
12–18 years	351,960 (35.241)	319,908 (34.663)	32,052 (42.291)	
Socioeconomic status				<0.001
Medical aid	43,190 (4.325)	28,841 (3.125)	14,349 (18.933)	
≤7 (Low)	179,037 (17.927)	164,914 (17.869)	14,123 (18.634)	
>7, ≤13	299,423 (29.981)	279,825 (30.319)	19,598 (25.858)	
>13, ≤16	182,771 (18.301)	170,505 (18.474)	12,266 (16.184)	
>16 (High)	281,624 (28.199)	266,548 (28.881)	15,076 (19.892)	
Missing	12,666 (1.268)	12,288 (1.331)	378 (0.499)	
Residence				<0.001
Urban	685,352 (68.624)	635,639 (68.873)	49,713 (65.593)	
Rural	313,228 (31.363)	287,151 (31.113)	26,077 (34.407)	
Missing	131 (0.013)	131 (0.014)	NA	
Medical comorbidities ^a^				
No	965,864 (96.711)	908,113 (98.396)	57,751 (76.199)	
Yes	32,847 (3.289)	14,808 (1.604)	18,039 (23.801)	
Chromosomal abnormality				<0.001
No	998,361 (99.965)	922,893 (99.997)	75,468 (99.575)	
Yes	350 (0.035)	28 (0.003)	322 (0.425)	
Cerebral palsy				<0.001
No	991,721 (99.300)	922,504 (99.955)	69,217 (91.327)	
Yes	6990 (0.700)	417 (0.045)	6573 (8.673)	
Health screening	498,626 (49.927)	481,722 (52.195)	16,904 (22.304)	<0.001
Follow-up duration				
(mean ± SD, years)	17.46 ± 2.315	18.00 ± 0.055	10.91 ± 4.921	<0.001

^a^ The composite variable was defined as having any of the following conditions: hypertension, diabetes, renal failure, rheumatoid arthritis, hypothyroidism, hyperthyroidism, hyperparathyroidism, chromosomal abnormality, or antiepileptic drugs-treated epilepsy. Individual conditions were analyzed separately in the unadjusted model. ID: Intellectual disability, SD: standard deviation, NA: not available.

**Table 2:** Hazard ratios for osteoporosis with concomitant fracture in children and adolescents with intellectual disability.

Variable	Unadjusted		Model 1		Model 2	
	HR	95%CI	HR	95%CI	HR	95%CI
ID status						
control	1 (Reference)		1 (Reference)		1 (Reference)	
ID	6.821	5.065–9.187	4.385	3.08–6.245	3.331	2.252–4.926
Sex						
Male	1 (Reference)		1 (Reference)		1 (Reference)	
Female	1.288	1.014–1.636	1.283	1.01–1.629	1.265	0.996–1.606
Age group						
2–6 years	1 (Reference)		1 (Reference)		1 (Reference)	
7–11 years	1.203	0.854–1.695	1.132	0.803–1.597	1.196	0.848–1.687
12–18 years	2.234	1.653–3.019	1.964	1.447–2.665	2.179	1.603–2.963
Socioeconomic status						
Medical aid	1 (Reference)		1 (Reference)		1 (Reference)	
≤7 (Low)	0.267	0.169–0.421	0.45	0.28–0.721	0.451	0.282–0.724
7 < , ≤ 13	0.255	0.168–0.387	0.458	0.296–0.709	0.463	0.299–0.716
13 < , ≤ 16	0.32	0.207–0.496	0.577	0.366–0.911	0.575	0.365–0.906
>16 (High)	0.305	0.203–0.459	0.523	0.34–0.802	0.517	0.337–0.793
Residential area						
Rural	1 (Reference)					
Urban	0.985	0.764–1.27				
Medical Comorbidities ^a^						
No	1 (Reference)		1 (Reference)		1 (Reference)	
Yes	5.501	3.839–7.883	2.353	1.561–3.547	1.797	1.157–2.791
Cerebral palsy						
No	1 (Reference)				1 (Reference)	
Yes	23.73	15.275–36.865			6.523	3.777–11.265

Adjustment models: Model 1: Adjusted for ID status, sex, age group, socioeconomic status, and medical comorbidities. Model 2: Adjusted for ID status, sex, age group, socioeconomic status, medical comorbidities, and cerebral palsy. ^a^ The composite variable was defined as having any of the following conditions: hypertension, diabetes, renal failure, rheumatoid arthritis, hypothyroidism, hyperthyroidism, hyperparathyroidism, chromosomal abnormality, or antiepileptic drugs-treated epilepsy. ID: Intellectual disability, SD: standard deviation; CI: confidence interval, HR: hazard ratio.

**Table 3:** Stratified analysis of osteoporosis-related fracture risk in children and adolescents with intellectual disability.

Variable	ID Group		Control Group		HR	95%CI	*p*-Value
	Number	Cases	Number	Cases			
Sex							
Male	49,034	40	590,170	122	7.597	5.225–11.045	<0.0001
Female	26,756	20	332,751	96	5.766	3.51–9.472	<0.0001
Age group							
2–6 years	19,737	14	303,480	47	8.188	4.354–15.399	<0.0001
7–11 years	24,001	19	299,533	54	9.501	5.479–16.476	<0.0001
12–18 years	32,052	27	319,908	117	4.859	3.157–7.479	<0.0001
Socioeconomic status							
Medical aid	14,349	20	28,841	13	7.057	3.219–15.473	<0.0001
Low	14,123	6	164,914	36	4.424	1.824–10.73	0.001
Medium-low	19,598	14	279,825	56	6.82	3.654–12.73	<0.0001
Medium-high	12,266	8	170,505	44	5.358	2.472–11.614	<0.0001
High	15,076	12	266,548	65	6.152	3.289–11.507	<0.0001
Residence							
Rural	31,632	39	287,151	25	8.882	5.218–15.117	<0.0001
Urban	44,158	21	635,639	193	4.579	2.892–7.249	<0.0001
Medical comorbidity							
No	57,751	32	908,113	211	5.045	3.448–7.38	<0.0001
Yes	18,039	28	14,808	7	5.624	2.332–13.561	0.0001
Cerebral palsy							
No	69,217	38	923,000	218	4.773	3.339–6.822	<0.0001
Yes	6573	22	417	0	NE ^a^	NE ^a^	0.9871

ID: Intellectual disability; CI: confidence interval, HR: hazard ratio. ^a^ NE: Not estimable due to zero events in the control group with cerebral palsy.

## Data Availability

The data that support the findings of this study are available from the National Health Insurance Service—National Health Information Database (NHIS-NHID); however, restrictions apply to the availability of these data, which were used under license for the current study, and so are not publicly available. Data are nonetheless available from the authors upon reasonable request and with permission of the NHIS.

## Supplementary Materials

The following supporting information can be downloaded at: https://www.mdpi.com/article/10.3390/medicina61101761/s1, Figure S1: Cumulative incidence of osteoporosis with pathologic fractures in the intellectual disability and control groups; Table S1: ICD-10 codes used to define fractures; Table S2: Clinical characteristics of the participants according to individual medical conditions; Table S3: Incidence rates of osteoporosis outcomes in children and adolescents with intellectual disability vs. control group; Table S4: Hazard ratios for osteoporosis with pathological fractures; Table S5: Distribution of osteoporosis diagnoses and anatomical sites of fractures.

## Abbreviations

The following abbreviations are used in this manuscript:

ID	intellectual disability
HR	hazard ratio
CI	confidence interval
CP	cerebral palsy
AED	antiepileptic drug
NHIS-NHID	National Health Insurance Service—National Health Information Database
DXA	dual-energy X-ray absorptiometry
ICD-10	International Classification of Diseases, 10th Revision
