# Supplementary material for: Risk of Osteoporosis-Related Fracture in Children and Adolescents with Intellectual Disability
Source: Medicina (Kaunas). 2025 Sep 29;61(10):1761. doi: 10.3390/medicina61101761 (PMC12566074; doi:10.3390/medicina61101761)
Supplement: Supplementary file 1 [file medicina-61-01761-s001.zip › medicina-3859618-supplementary.pdf]

Table S1. ICD-10 codes used to define fractures

Fracture Sites	ICD-10 Code
Rib, sternum, thoracic spine	S22.x
Lumbar spine, pelvis	S32.x
Scapular/clavicle/humerus	S42.x
Forearm	S52.x
Wrist and hand	S62.0x,S62.8x
Femur	S72.x
Lower limb (including ankle)	S82.x
Collapsed vertebra, not elsewhere classified	M48.5x

ICD-10, International Classification of Diseases, 10th Revision

Table S2. Clinical characteristics of the participants according to individual medical conditions

Characteristics	Original cohort			*P*-value
	TOTAL	Control group	Intellectual disability group	
	998,711 (100%)	922,921 (92.4%)	75,790 (7.6%)	
Medical comorbidities[a]				<0.001
No	965,864 (96.711)	908,113 (98.396)	57,751 (76.199)	
Yes	32,847 (3.289)	14,808 (1.604)	18,039 (23.801)	
Hypertension				<0.001
No	996,805 (99.809)	922,026 (99.903)	74,779 (98.666)	
Yes	1,906 (0.191)	895 (0.097)	1,011 (1.334)	
Diabetes				<0.001
No	995,765 (99.705)	921,559 (99.852)	74,206 (97.910)	
Yes	2,946 (0.295)	1,362 (0.148)	1,584 (2.090)	
Renal failure				<0.001
No	998,318 (99.961)	922,763 (99.983)	75,555 (99.690)	
Yes	393 (0.039)	158 (0.017)	235 (0.310)	
Rheumatoid arthritis				<0.001
No	997,646 (99.893)	922,285 (99.931)	75,361 (99.434)	
Yes	1,065 (0.107)	636 (0.069)	429 (0.566)	
Hypothyroidism				<0.001
No	995,763 (99.705)	922,296 (99.932)	73,467 (96.935)	
Yes	2,948 (0.295)	625 (0.068)	2,323 (3.065)	
Hyperthyroidism				<0.001
No	997,631 (99.892)	922,325 (99.935)	75,306 (99.361)	
Yes	1,080 (0.108)	596 (0.065)	484 (0.639)	
Hyperparathyroidism				<0.001
No	998,695 (99.998)	922,918 (100.00)	75,777 (99.983)	
Yes	16 (0.002)	3 (0.000)	13 (0.017)	
AED-treated epilepsy				<0.001
No	983,215 (98.448)	920,657 (99.755)	62,558 (82.541)	
Yes	15,496 (1.552)	2,264 (0.245)	13,232 (17.459)	
Chromosomal abnormality				<0.001
No	998,361 (99.965)	922,893 (99.997)	75,468 (99.575)	
Yes	350 (0.035)	28 (0.003)	322 (0.425)	
Cerebral palsy				<0.001
No	991,721 (99.300)	922,504 (99.955)	69,217 (91.327)	

Yes	6,990 (0.700)	417 (0.045)	6,573 (8.673)

[a] The composite variable was defined as having any of the following conditions: hypertension, diabetes, renal failure, rheumatoid arthritis, hypothyroidism, hyperthyroidism, hyperparathyroidism, chromosomal abnormality, or AED-treated epilepsy. Individual conditions were analyzed separately in the unadjusted model.

AED: antiepileptic drug

Table S3. Incidence rates of osteoporosis outcomes in children and adolescents with intellectual disability vs. control group

Outcome	Control group			ID group			IRR (95% CI)	P-value
	n	Cases	IR*	n	Cases	IR[a]		
Osteoporosis with concomitant fracture	922,921	218	0.013	75,790	60	0.072	5.47 (4.10-7.29)	<0.001
Osteoporosis with pathological fracture	922,921	156	0.009	75,790	50	0.060	6.44 (4.68-8.85)	<0.001

ID: intellectual disability; IRR: incidence rate ratio; CI: confidence interval

[a]IR: Incidence rate per 1,000 person-years

Table S4. Hazard ratios for osteoporosis with pathological fractures

	Unadjusted		Model 1		Model 2	
	HR	95% CI	HR	95% CI	HR	95% CI
Case						
Control	1 (Reference)		1 (Reference)		1 (Reference)	
ID	7.420	5.338–10.313	4.730	3.186–7.021	3.439	2.207–5.358
Sex						
Female	1.223	0.927–1.615	1.221	0.924–1.612	1.199	0.908–1.584
Male						
Age group						
2–6 years	1 (Reference)		1 (Reference)		1 (Reference)	
7–11 years	1.460	1.003–2.124	1.351	0.927–1.968	1.444	0.990–2.106
12–18 years	1.878	1.319–2.673	1.602	1.120–2.290	1.823	1.271–2.614
Socioeconomic status						
Medical aid						
<=7 (Low)	0.345	0.200–0.594	0.612	0.348–1.074	0.616	0.351–1.082
7<, <=13	0.312	0.188–0.518	0.578	0.34–0.983	0.587	0.345–0.998
13<, <=16	0.345	0.201–0.592	0.635	0.362–1.116	0.633	0.361–1.111
>16 (High)	0.406	0.248–0.665	0.729	0.434–1.226	0.722	0.430–1.211
Residence						
Rural	1 (Reference)		1 (Reference)		1 (Reference)	
Urban	1.054	0.783–1.419	1.073	0.795–1.448	1.053	0.780–1.420
Medical comorbidities[a]						
No	1 (Reference)		1 (Reference)		1 (Reference)	
Yes	6.730	4.589–9.870	2.832	1.815–4.421	7.037	3.929–12.600
Cerebral palsy						
No	1 (Reference)					
Yes	28.756	18.207–45.416				

Adjustment models:

Model 1: adjusted for age, sex, socioeconomic status, residence, and medical comorbidities[a]
Model 2: adjusted for age, sex, socioeconomic status, residence, medical comorbidities[a], and cerebral palsy
[a] The composite variable was defined as having any of the following conditions: hypertension, diabetes, renal failure, rheumatoid arthritis, hypothyroidism, hyperthyroidism, hyperparathyroidism, chromosomal abnormality, or antiepileptic drugs-treated epilepsy. ID: Intellectual disability, CI: confidence interval, HR: hazard ratio

Table S5. Distribution of osteoporosis diagnoses and anatomical sites of fractures

Characteristics	Total	Control Group	Intellectual disability Group	*P* - value
	998,711 (100%)	922,921 (92.4%)	75,790 (7.6%)	
Osteoporosis in total				
No	993,125 (99.441)	918,050 (99.472)	75,075 (99.057)	<0.001
Yes	5,586 (0.559)	4,871 (0.528)	715 (0.943)	<0.001
Osteoporosis with concomitant fracture				
No	998,433 (99.972)	922,703 (99.976)	75,730 (99.921)	<0.001
Yes	278 (0.028)	218 (0.024)	60 (0.079)	<0.001
Osteoporosis with pathological fracture				
No	998,505 (99.979)	922,765 (99.983)	75,740 (99.934)	<0.001
Yes	206 (0.021)	156 (0.017)	50 (0.066)	<0.001
Rib, sternum, thoracic spine				
No	981,760 (98.303)	906,483 (98.219)	75,277 (99.323)	<0.001
Yes	16,951 (1.697)	16,438 (1.781)	513 (0.677)	<0.001
Lumbar spine, pelvis				
No	987,445 (98.872)	912,234 (98.842)	75,211 (99.236)	<0.001
Yes	11,266 (1.128)	10,687 (1.158)	579 (0.764)	<0.001
Scapular/clavicle/humerus				
No	969,338 (97.059)	897,051 (97.197)	72,287 (95.378)	<0.001
Yes	29,373 (2.941)	25,870 (2.803)	3,503 (4.622)	<0.001
Forearm				
No	929,520 (93.072)	857,806 (92.945)	71,714 (94.622)	<0.001
Yes	69,191 (6.928)	65,115 (7.055)	4,076 (5.378)	<0.001
Wrist/hand				
No	991,003 (99.228)	915,475 (99.193)	75,528 (99.654)	<0.001

Yes	7,708 (0.772)	7,446 (0.807)	262 (0.346)	<0.001
Femur				
No	995,572 (99.686)	920,386 (99.725)	75,186 (99.203)	<0.001
Yes	3,139 (0.314)	2,535 (0.275)	604 (0.797)	<0.001
Lower limb (including ankle)				
No	958,233 (95.947)	885,742 (95.972)	72,491 (95.647)	<0.001
Yes	40,478 (4.053)	37,179 (4.028)	3,299 (4.353)	<0.001
Collapsed vertebra, not elsewhere classified				
No	998,593 (99.988)	922,809 (99.988)	75,784 (99.992)	0.304
Yes	118 (0.012)	112 (0.012)	6 (0.008)	0.304

Figure S1. Cumulative incidence of osteoporosis with pathologic fractures in the intellectual disability and control groups

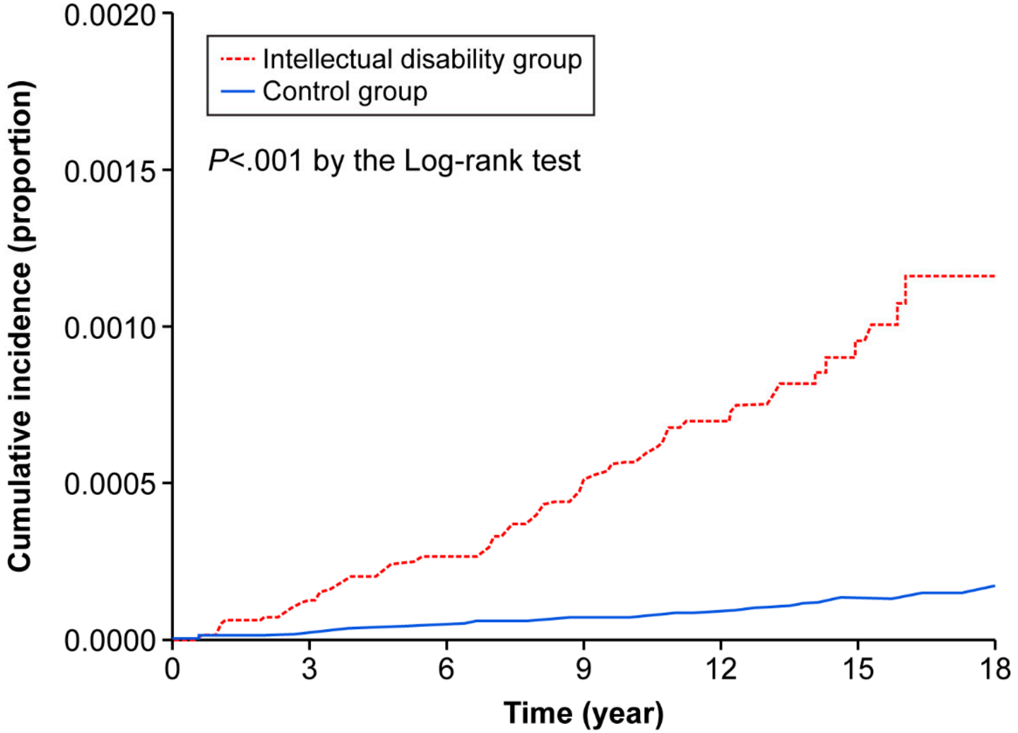

Intellectual disability group

75790 68057 60252 50455 36961 19184 0

Control group

922918 922898 922876 922854 922836 922797 0
